# Impact of thyroid function on cystatin C in detecting acute kidney injury: a prospective, observational study

**DOI:** 10.1186/s12882-019-1201-9

**Published:** 2019-02-06

**Authors:** Danqing Zhang, Lu Gao, Heng Ye, Ruibin Chi, Lin Wang, Linhui Hu, Xin Ouyang, Yating Hou, Yujun Deng, Yi Long, Weiping Xiong, Chunbo Chen

**Affiliations:** 1 Department of Intensive Care Unit of Cardiac Surgery, Guangdong Cardiovascular Institute, Guangdong Provincial People’s Hospital, Guangdong Academy of Medical Sciences, 96 Dongchuan Road, Guangzhou, 510080 Guangdong Province People’s Republic of China; 2 Department of Critical Care, Guangdong Provincial People’s Hospital, Guangdong Academy of Medical Sciences, 106 Zhongshan Er Road, Guangdong, 510080 Guangdong Province People’s Republic of China; 3 Shantou University Medical College, 22 Xinling Road, Shantou, 515063 Guangdong Province People’s Republic of China; 4 Department of Critical Care Medicine, Guangzhou Nansha Central Hospital, Guangzhou, 511400 Guangdong Province People’s Republic of China; 5 Department of Critical Care Medicine, Xiaolan Hospital of Southern Medical University, Zhongshan, 528415 Guangdong Province People’s Republic of China; 6 National Clinical Research Center for Kidney Disease, State Key Laboratory of Organ Failure Research, Nanfang Hospital, Southern Medical University, Guangzhou, 510515 Guangdong Province People’s Republic of China

**Keywords:** Acute kidney injury, Cystatin C, Intensive care unit, Renal biomarker, Thyroid function

## Abstract

**Background:**

Cystatin C (Cys C) used clinically for detecting early acute kidney injury (AKI) was reported to be associated with thyroid function. Therefore, whether the performance of Cys C is affected by thyroid hormones has raised concern in critically ill patients. This study aimed to investigate the impact of thyroid hormones on the diagnostic and predictive accuracy of Cys C for AKI, and hence optimize the clinical application of Cys C.

**Methods:**

A prospective observational study was conducted in the general intensive care units (ICUs). Serum creatinine (SCr), Cys C, and thyroid function were documented for all patients at ICU admission. Patients were separated into five quintiles based on free triiodothyronine (FT3) and total triiodothyronine (TT3), and two categories according to the presence of low T3 syndrome or not. The impact of thyroid function on the performance of Cys C in diagnosing and predicting AKI was assessed by area under the receiver operating characteristic curve (AUC).

**Results:**

The AKI incidence was 30.0% (402/1339); 225 patients had AKI upon entry, and 177 patients developed AKI during the subsequent 7 days. The AUCs for Cys C in detecting total AKI, established AKI, and later-onset AKI was 0.753, 0.797, and 0.669, respectively. The multiple linear regression analysis demonstrated that TT3 and FT3 were independently associated with Cys C. Overall, although Cys C did not yield any significant difference in AUCs for detecting AKI among patients with different thyroid hormones, the optimal cut-off value of Cys C to detect AKI was markedly different between patients with and without low T3 syndrome.

**Conclusions:**

The thyroid function had no significant impact on the diagnostic and predictive accuracy of Cys C in detecting AKI in ICU patients. However, the optimal cut-off value of Cys C to detect AKI could be affected by thyroid function.

**Electronic supplementary material:**

The online version of this article (10.1186/s12882-019-1201-9) contains supplementary material, which is available to authorized users.

## Key messages

- The thyroid hormones at admission were negatively associated with Cys C, and only TT3 and FT3 were independent factors correlated with Cys C.
- Thyroid function alteration, including low T3 syndrome, might not exert a statistically significant impact on the performance of Cys C in diagnosing and predicting AKI in ICU patients.
- The optimal cut-off value of Cys C to detect AKI could be affected by thyroid function.

## Background

Acute kidney injury (AKI) is an increasingly prevalent problem in patients hospitalized in intensive care units (ICUs), with the incidence varied from 9% to more than 50% [1, 2]. Despite the development of renal replacement therapy (RRT), AKI remains a major cause of adverse outcomes, including high mortality, increased length of hospital and ICU stay, as well as the economic burden [3, 4]. Timely detection of AKI is important to prevent its progression and hence potentially improve the timeliness of intervention. Nevertheless, the diagnosis of AKI based on conventional surrogate markers, serum creatinine (SCr), and urine output lags far behind the reduction in kidney function [5, 6]. Although some novel biomarkers are clinically available, the application of these biomarkers still has limitations [7]. In this regard, the optimal utilization of the available biomarker would be a tremendous advance in clinical medicine.

Serum cystatin C (Cys C) has been reported to be a clinically available marker for early detecting AKI, which is routinely used in some hospitals [7–9]. Cys C is a cysteine proteinase inhibitor produced at a constant rate in all nucleated cells and minimally bound to proteins. It is freely filtered by the glomerulus and completely reabsorbed by the proximal tubules [10, 11]. Compared with SCr, the concentration of serum Cys C appeared to be less affected by muscle mass, diet, age, and sex, allowing for the superiority of Cys C to be a reliable marker for detecting an early minimal reduction in the glomerular filtration rate (GFR) [12–15]. However, growing evidence has demonstrated that the production of Cys C could be stimulated by thyroid hormones, and it was sensitive to small changes in thyroid function [16–18]. Although other studies proved that the performance of Cys C in the diagnosis and risk prediction of AKI was not impacted by thyroid function [19, 20], these results have not been verified in larger studies.

Therefore, this prospective, observational study was conducted in a larger critically ill cohort to analyze the association between thyroid function and the diagnostic and predictive accuracy of Cys C in detecting AKI.

## Methods

### Study design and participants

This prospective observational study was conducted in the general ICUs in Guangdong General Hospital. All consecutive patients aged 18 years or elder admitted to ICUs from October 2014 to December 2016 were enrolled for the study. The exclusion criteria included refusal of consent, preexisting hyperthyroidism or hypothyroidism, medical history of hormone replacement therapy except insulin use, preexisting end-stage renal disease (ESRD) or renal dysfunction requiring RRT before admission, preexisting renal transplantation or nephrectomy, or missing admission data. The primary outcome of this study was the detection of AKI within 1 week after ICU admission, and the secondary outcome comprised length of ICU and hospital stay, RRT during ICU stay, as well as ICU and hospital mortality.

### Data collection and definition

Each patient’s baseline characteristics, including age, gender, body mass index (BMI), preexisting clinical conditions, sepsis, admission type, baseline SCr, baseline estimated glomerular filtration rate (eGFR), acute physiology and chronic health evaluation (APACHE II) score, length of ICU stay, length of hospital stay, RRT during ICU stay, ICU mortality, and hospital mortality, were collected from the electronic medical system. The serum sample for measuring SCr, Cys C, total triiodothyronine (TT3, normal value range: 1.34–2.73 nmol/L), total thyroxine (TT4, normal value range: 78.40–158.40 nmol/L), free triiodothyronine (FT3, normal value range: 3.80–6.00 pmol/L), free thyroxine (FT4, normal value range: 7.50–21.10 pmol/L), thyroid-stimulating-hormone (TSH; normal value range: 0.34–5.60 μIU/mL), blood urea nitrogen (BUN), and albumin was collected at admission, and thereafter SCr was measured daily between 7 a.m. to 9 a.m. as a part of routine clinical care until ICU discharge. The eGFR was calculated using the Chronic Kidney Disease Epidemiology Collaboration (CKD-EPI) creatinine equation [21].

A baseline SCr was determined using the following rules ranked in the descending order of preference [22]: (1) the most recent pre-ICU value between 30 and 365 days before ICU admission (*n* = 143); (2) a stable pre-ICU value > 365 days for patients aged < 40 years (stable defined as within 15% of the lowest ICU measurement) before ICU admission (*n* = 6); (3) pre-ICU value > 365 days before ICU admission and less than the initial SCr at ICU admission (*n* = 51, 4) a pre-ICU value (between 3 and 39 days before ICU admission) less than or equal to the initial SCr on admission to ICU and not distinctly in AKI (*n* = 638); or (5) the lowest SCr upon initial admission to ICU (*n* = 173), the last ICU value (*n* = 216), or the minimum value at follow-up up to 365 days (*n* = 112). According to the KDIGO (Kidney Disease: Improving Global Outcomes) criteria, patients with an increase in SCr by ≥0.3 mg/dL (≥26.5 μmol/L) within 48 h, or increase in SCr to ≥1.5 times baseline within 1 week, or urine output < 0.5 mL/(kg ·h) for 6 h were diagnosed with AKI. However, since the urine output criteria has limited sensitivity when diuretics are administrated [23], the AKI diagnosis was based on serum creatinine in this study. Established AKI was defined as the diagnosis of AKI on entry, and later-onset AKI indicated no AKI diagnosis on entry but meeting the KDIGO criteria during the following 1 week after admission. Low T3 syndrome, also named non-thyroidal illness syndrome, is characterized by decreased serum T3 and, in severe illness, decreased serum T4, increased serum reverse T3 (rT3) concentrations, and normal or slightly decreased concentration of TSH [24, 25]. In this study, patients with an FT3 level below normal lower bound, and FT4 and TSH lower or within the normal range were diagnosed with low T3 syndrome according to the manufacturer’s standard value, as described in a previous study [26].

### Laboratory methods

All samples were collected within 1 h after ICU admission and measured within 24 h after collection. SCr, serum Cys C, albumin, and BUN were measured using a UniCel DxC 800 Synchron System (Beckman Coulter, CA, USA). Cys C was assayed by immunoturbidimetry, the coefficients of interassay and intraassay variations for which were ≤ 5% and ≤ 10%, respectively. The thyroid function test was conducted via chemiluminescent immunoassay using Unicel DxI800 Synchron System (Beckman Coulter, CA, USA).

### Statistical analysis

All statistical analyses were conducted using SPSS version 16.0 (SPSS, IL, USA; 2007), and MedCalc version 15.8 (MedCalc Software bvba, Ostend, Belgium; 2015) software programs. A two-tailed *P* value *<* 0.05 was considered statistically significant. The characteristics of patients with continuous and categorical data were presented with median (interquartile range, IQR) and absolute value (percentage), respectively. For continuous variables, normally distributed variables were compared using the *t* test and non-normally distributed variables were compared using the Wilcoxon rank-sum test. Categorical variables were compared using the chi-square test or Fisher’s exact test. The bivariate correlation analysis was used to evaluate the association between two variables. A multivariable linear regression analysis was conducted to identify the independent factors for Cys C and determine the representative index of thyroid function for further analysis. A receiver operating characteristic (ROC) curve analysis was performed, and the area under the curve (AUC) was calculated to demonstrate the diagnostic and predictive value of Cys C in detecting AKI. The difference between AUCs in each group was calculated using the Hanley–McNeil method [27], and the optimal cutoff value for AKI detection was determined with the Youden’s index using the MedCalc software.

## Results

### Patient characteristics and outcomes

**Fig. 1:**
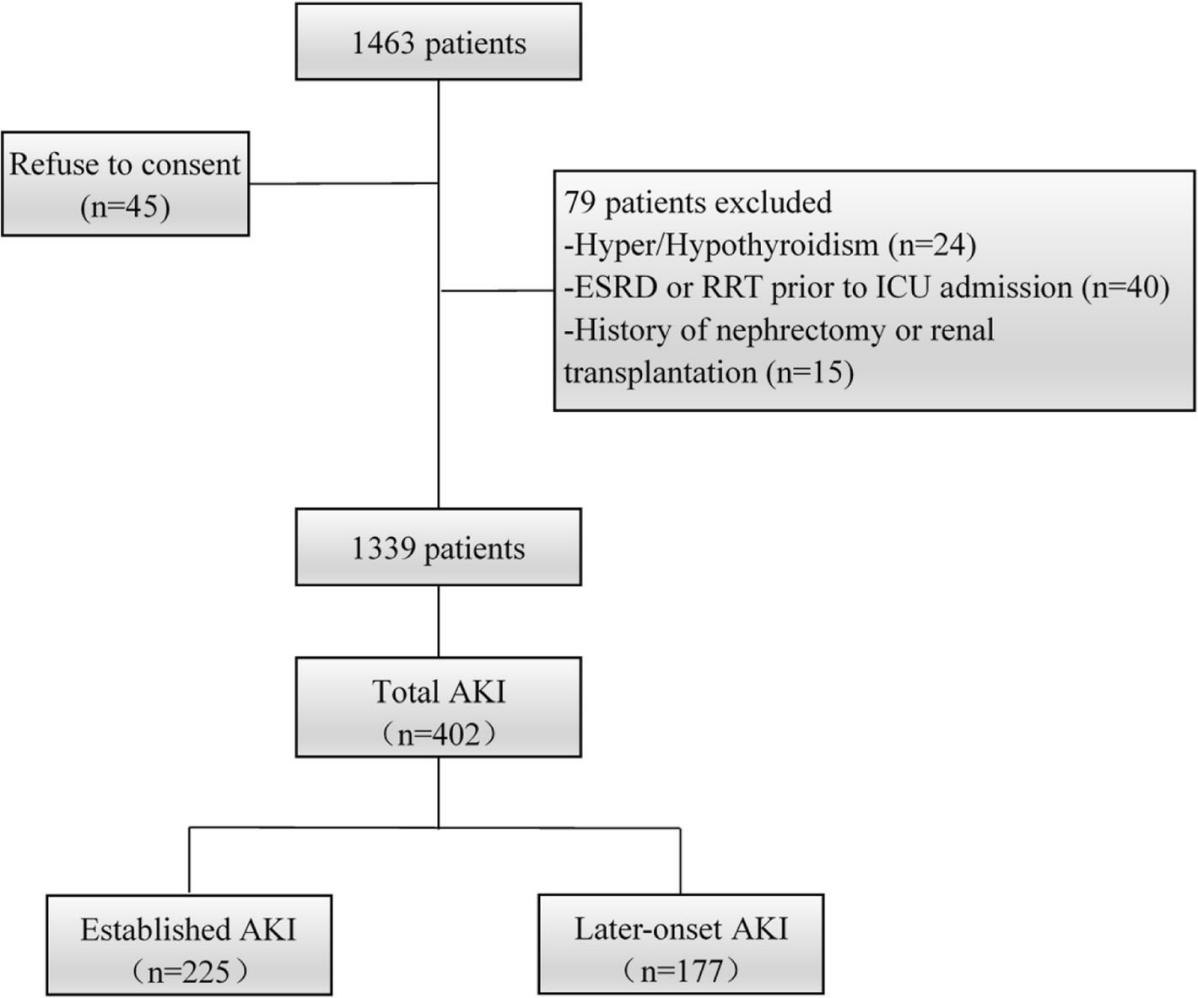
Flow diagram of the selection process. AKI, acute kidney injury; ESRD, end stage renal disease; RRT, renal replacement therapy

Figure 1 presents the protocol and flow diagram of screening process. Among 1463 critically ill adult patients enrolled for the study, 124 were excluded for the following reasons: refused to consent (*n* = 45), complicated with thyroid dysfunction (hyperthyroidism or hypothyroidism, *n* = 24), history of nephrectomy (*n* = 10) or renal transplantation (*n* = 5), and ESRD (*n* = 40) prior to ICU admission. Finally, 1339 patients got involved in the study; AKI occurred in 402 patients, of which 225 were diagnosed with AKI at admission and 177 developed AKI during the following 1 week.

Basic clinical data and outcomes of the patients were demonstrated in Table 1. Compared with non-AKI patients, the patients with AKI were elder and had a significantly higher rate of complications, including hypertension (HTN), diabetes mellitus (DM), chronic kidney disease (CKD), coronary artery disease (CAD), chronic heart failure (CHF), stoke, and chronic liver disease. Sepsis was significantly associated with AKI, with an occurrence rate of 33.1%. The thyroid hormones concentration was significantly lower in the AKI group than in the non-AKI group, and patients in AKI group tended to be complicated with low T3 syndrome. Emergency patients and medical patients admitted to ICU were predisposed to be complicated with AKI. Patients with AKI showed significantly higher hospital and ICU mortality, and longer hospital and ICU stay.

**Table 1:** Baseline clinical data and outcomes^a^

Characteristics	Non-AKI (*n* = 937)	AKI (*n* = 402)	*P* value
Demographic variables			
Age, years	52.0 (40.2–62.3)	61.0 (47.0–71.0)	< 0.001
Male sex, *n* (%)	475 (50.7)	233 (58.0)	0.017
BMI, kg/m^2^	22.19 (20.70–23.44)	22.16 (20.57–23.11)	0.477
Preexisting clinical conditions			
Hypertension, *n* (%)	113 (12.1)	123 (30.6)	< 0.001
DM, *n* (%)	41 (4.4)	62 (15.4)	< 0.001
CKD, *n* (%)	16 (1.7)	51 (12.7)	< 0.001
CAD, *n* (%)	17 (1.8)	27 (6.7)	< 0.001
Stroke, *n* (%)	93 (9.9)	93 (23.1)	< 0.001
CHF, *n* (%)	10 (1.1)	25 (6.2)	< 0.001
Malignancy, *n* (%)	113 (12.1)	64 (15.9)	0.064
COPD, *n* (%)	15 (1.6)	13 (3.2)	0.062
Chronic Liver disease, *n* (%)	4 (0.4)	8 (2.0)	0.009
Sepsis, n (%)	76 (8.1)	133 (33.1)	< 0.001
Admission type, *n* (%)			< 0.001
Elective surgical, *n* (%)	821 (87.6)	236 (58.7)	
Emergency surgical, *n* (%)	51 (5.4)	69 (17.2)	
Medical, *n* (%)	65 (6.9)	97 (24.1)	
Baseline serum creatinine, mg/dl	0.69 (0.58–0.82)	0.70 (0.57–0.94)	0.053
Baseline eGFR, ml/minute/1.73 m^2^	104.58 (93.69–115.48)	98.53 (79.57–112.31)	< 0.001
Serum creatinine at admission, mg/dl	0.76 (0.64–0.92)	1.02 (0.77–1.39)	< 0.001
Cys C at admission, mg/L	0.74 (0.59–0.91)	1.04 (0.77–1.51)	< 0.001
BUN at admission,mg/dl	11.09 (8.88–13.73)	15.13 (10.50–26.05)	< 0.001
Albumin at admission, g/L	31.70 (28.00–35.00)	30.50 (25.85–34.74)	< 0.001
Thyroid function at admission			
FT3 (pmol/L)	3.88 (3.39–4.32)	3.52 (2.86–4.04)	< 0.001
TT3 (nmol/L)	0.99 (0.81–1.18)	0.81 (0.58–1.05)	0.001
FT4 (pmol/L)	13.37 (11.38–15.43)	13.16 (10.86–15.66)	0.436
TT4 (nmol/L)	99.80 (84.30–114.53)	93.80 (71.58–110.82)	< 0.001
TSH (μIU/L)	1.36 (0.72–2.37)	0.92 (0.52–1.86)	< 0.001
Low T3 syndrome at admission, *n* (%)	404 (43.1)	246 (61.2)	< 0.001
APACHE II score	9 (7–13)	15 (10–24)	< 0.001
UP, ml/kg/h	2.12 (1.61–2.80)	2.00 (1.39–2.78)	0.008
Outcomes			
Length of ICU stay, days	2 (2–4)	4 (2–9)	< 0.001
Length of hospital stay, days	15 (12–21)	18 (13–28)	< 0.001
RRT during ICU stay, *n* (%)	3 (0.3)	17 (4.2)	< 0.001
ICU mortality, *n* (%)	7 (0.7)	34 (8.5)	< 0.001
In-hospital mortality, *n* (%)	8 (0.9)	39 (9.7)	< 0.001

^**a**^The non-normally distributed continuous variables are expressed as median (25th percentile to 75th percentile [interquartile range]). Categorical variables are expressed as n (%). *APACHE II* Acute Physiology and Chronic Health Evaluation score, *AKI* acute kidney injury, *BMI* body mass index, *BUN* blood urea nitrogen, *CKD* chronic kidney disease, defined as baseline estimated glomerular filtration rate < 60 ml/min/1.73 m^2^, *Cys C* cystatin C, *DM* diabetes mellitus, *eGFR* estimated glomerular filtration rate, *FT3* free triiodothyronine, *FT4* free thyroxine, *ICU* intensive care unit, *RRT* renal replacement therapy, *TSH* thyroid-stimulating-hormone, *TT3* total triiodothyronine, *TT4* total thyroxine, *UP* urine production first 24 h after admission. P value for global comparisons among groups by *t* or Kruskal-Wallis test, and chi-square test for continuous and categorical variables, respectively

### Factors associated with Cys C

As indicated in Table 2 showing the bivariate correlation analysis between Cys C and other factors, patients with a higher Cys C concentration were elder and had higher APACHE II score, SCr at admission, baseline SCr, and BUN. Moreover, albumin, and thyroid hormone concentrations, including FT3, TT3, and TT4 were negatively associated with Cys C. The multiple linear regression analysis shown in Table 3 indicated that among the abovementioned thyroid hormones, only FT3 (standardized *β* = − 0.100, *P* < 0.001) and TT3 (standardized *β* = 0.059, *P* = 0.007) were independently associated with Cys C. In addition, sex (standardized *β* = − 0.059, *P* < 0.001), age (standardized *β* = 0.136, *P* < 0.001), APACHE II score (standardized *β* = 0.087, *P* < 0.001), SCr at admission (standardized *β* = 0.578, *P* < 0.001), baseline SCr (standardized *β* = 0.066, *P* = 0.004), and BUN at admission (standardized *β* = 0.166, *P* < 0.001) were independent factors related to Cys C.

**Table 2:** Factors associated with Cys C using bivariate correlation analysis

Spearman’s rho	Cys C (mg/L)	
	R	*P*
Age, years	0.380	< 0.001
Male sex	0.262	< 0.001
Baseline serum creatinine (mg/dl)	0.392	< 0.001
Serum Creatinine at admission (mg/dl)	0.546	< 0.001
BUN at admission (mg/dl)	0.471	< 0.001
Albumin at admission (g/L)	- 0.127	< 0.001
FT3 (pmol/L)	- 0.257	< 0.001
TT3 (nmol/L)	- 0.189	< 0.001
FT4 (pmol/L)	- 0.050	0.065
TT4 (nmol/L)	- 0.122	< 0.001
TSH (μIU/L)	- 0.032	0.242
APACHE II Score	0.348	< 0.001

*APACHE II score* Acute Physiology and Chronic Health Evaluation II score, *BUN* blood urea nitrogen, *Cys C* cystatin C, *FT3* free triiodothyronine, *FT4* free thyroxine, *TSH* thyroid-stimulating-hormone, *TT3* total triiodothyronine, *TT4* total thyroxine

**Table 3:** Factors associated with Cys C using multivariate linear regression analysis^a^

Variables	Cys C (mg/L)	
	Standardized β	*P*
Age, years	0.136	< 0.001
Male sex	- 0.059	< 0.001
Baseline Serum creatinine (mg/dl)	0.066	0.004
Serum creatinine at admission (mg/dl)	0.578	< 0.001
BUN at admission (mg/dl)	0.166	< 0.001
FT3 (pmol/L)	−0.100	< 0.001
TT3 (nmol/L)	0.059	0.007
APACHE II Score	0.087	< 0.001
Constant	0.116 [unstandardized]	0.043

^a^Independent variables including age, male sex, APACHE II Score, serum creatinine at admission, baseline serum creatinine, BUN at admission, albumin at admission, FT3, TT3, and TT4 were involved in the stepwise analysis. Adjusted R square was 0.681. APACHE II score, Acute Physiology and Chronic Health Evaluation II score, *BUN* blood urea nitrogen, *Cys C* cystatin C, *FT3* free triiodothyronine, *FT4* free thyroxine, *TT3* total triiodothyronine, *TT4* total thyroxine

### Detection of AKI using Cys C with respect to thyroid hormones stratification

**Fig. 2:**
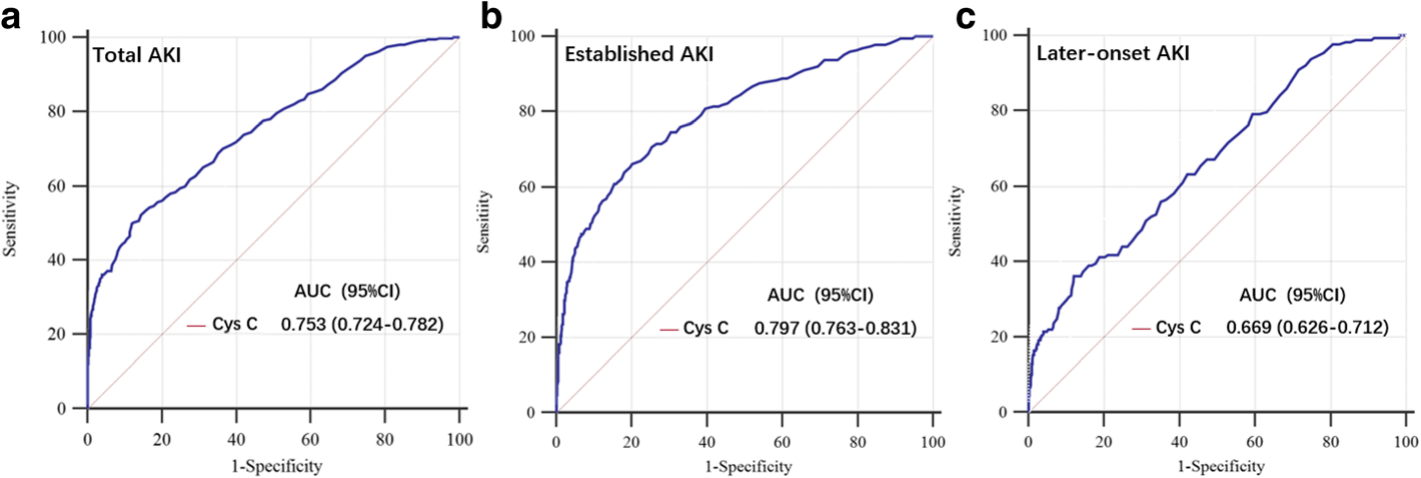
ROC analysis of Cys C for AKI detection. Among 1339 adult critically ill patients, 402 (30.0%) were diagnosed with AKI (**a** Total AKI). Of 402 patients with AKI, 225 patients were diagnosed with established AKI (**b** Established AKI) and 177 were diagnosed with later-onset AKI (**c** Later-onset AKI). AKI, acute kidney injury; Cys C, cystatin C

Cys C was significantly higher in the AKI group than the non-AKI group (Table 1). Figure 2 demonstrated the ROC analysis for the diagnostic and predictive ability of Cys C in detecting AKI. The AUCs calculated for Cys C was 0.753, 0.797, and 0.669 in detecting total AKI, established AKI, and later-onset AKI, respectively.

The patients were divided into five quintiles based on FT3 (Table 4), TT3 (Table 5), and FT4 (Additional file 1: Table S1), and AUC-ROC analysis was conducted to evaluate the impact of thyroid hormones on Cys C in detecting AKI. In the stratification of FT3 (Table 4), AUCs for Cys C in detecting total AKI, established AKI, and later-onset AKI was 0.777, 0.786, and 0.667, respectively, in quintile I; 0.740, 0.799, and 0.658, respectively, in quintile II; 0.721, 0.774, and 0.617, respectively, in quintile III; 0.727, 0.751, and 0.694, respectively, in quintile IV; and 0.700, 0.733, and 0.661, respectively, in quintile V. For quintiles of TT3 (Table 5), Cys C demonstrated its diagnostic value as indicated by AUCs of 0.784, 0.738, 0.730, 0.708, and 0.707, respectively, to detect total AKI; 0.804, 0.781, 0.786, 0.706, and 0.757, respectively, to detect established AKI; and 0.651, 0.652, 0.657, 0.690, and 0.670, respectively, to detect later-onset AKI. In summary, despite a tendency of decreasing AUCs across the quintiles by FT3 and TT3 in total and established AKI, no significant difference between AUCs was observed in any two groups for total AKI, established AKI, and later-onset AKI in different stratification of thyroid hormones.

**Table 4:** Detection of AKI using Cys C by quintiles of FT3

	Total AKI^a^				Established AKI^b^				Later-onset AKI^c^			
	AUC ROC	95%CI	Cut-off	*P*	AUC ROC	95%CI	Cut-off	*P*	AUC ROC	95%CI	Cut-off	*P*
Total	0.753 ± 0.015	0.724–0.782	1.03	< 0.001	0.797 ± 0.017	0.763–0.831	0.98	< 0.001	0.669 ± 0.022	0.626–0.712	1.03	< 0.001
I (n = 268)	0.777 ± 0.028	0.722–0.832	1.24	< 0.001	0.786 ± 0.030	0.727–0.845	1.22	< 0.001	0.667 ± 0.050	0.570–0.764	1.21	0.001
II (*n* = 268)	0.740 ± 0.035	0.671–0.809	0.98	< 0.001	0.799 ± 0.045	0.712–0.887	1.10	< 0.001	0.658 ± 0.048	0.564–0.751	0.71	0.003
III (*n* = 267)	0.721 ± 0.037	0.648–0.793	0.99	< 0.001	0.774 ± 0.042	0.691–0.857	0.90	< 0.001	0.617 ± 0.057	0.505–0.729	1.04	0.049
IV (*n* = 268)	0.727 ± 0.035	0.658–0.795	0.78	< 0.001	0.751 ± 0.049	0.655–0.846	0.82	< 0.001	0.694 ± 0.044	0.608–0.780	0.60	< 0.001
V (*n* = 268)	0.700 ± 0.039	0.624–0.776	0.79	< 0.001	0.733 ± 0.049	0.637–0.829	0.79	0.001	0.661 ± 0.052	0.559–0.762	1.03	0.003

^a^For Total AKI, Quintile I versus Quintile II Z = 0.825, *P* = 0.409; Quintile I versus Quintile III Z = 1.207, *P* = 0.227; Quintile I versus Quintile IV Z = 1.116, *P* = 0.265; Quintile I versus Quintile V Z = 1.604, *P* = 0.109; Quintile II versus Quintile III Z = 0.373, *P* = 0.709; Quintile II versus Quintile IV Z = 0.263, *P* = 0.793; Quintile II versus Quintile V Z = 0.763, *P* = 0.445; Quintile III versus Quintile IV Z = 0.118, *P* = 0.906; Quintile III versus Quintile V Z = 0.391, *P* = 0.696; Quintile IV versus Quintile V Z = 0.515, *P* = 0.606. ^b^For Established AKI, Quintile I versus Quintile II Z = 0.240, *P* = 0.810; Quintile I versus Quintile III Z = 0.232, *P* = 0.816; Quintile I versus Quintile IV Z = 0.609, *P* = 0.542; Quintile I versus Quintile V Z = 0.922, *P* = 0.356; Quintile II versus Quintile III Z = 0.406, *P* = 0.685; Quintile II versus Quintile IV Z = 0.721, *P* = 0.471; Quintile II versus Quintile V Z = 0.992, *P* = 0.321; Quintile III versus Quintile IV Z = 0.356, *P* = 0.721; Quintile III versus Quintile V Z = 0.635, *P* = 0.525; Quintile IV versus Quintile V Z = 0.260, *P* = 0.795. ^c^For Later-onset AKI, Quintile I versus Quintile II Z = 0.130, *P* = 0.897; Quintile I versus Quintile III Z = 0.660, *P* = 0.510; Quintile I versus Quintile IV Z = 0.405, *P* = 0.685; Quintile I versus Quintile V Z = 0.083, *P* = 0.934; Quintile II versus Quintile III Z = 0.550, *P* = 0.582; Quintile II versus Quintile IV Z = 0.553, *P* = 0.580; Quintile II versus Quintile V Z = 0.042, *P* = 0.966; Quintile III versus Quintile IV Z = 1.069, *P* = 0.285; Quintile III versus Quintile V Z = 0.570, *P* = 0.568; Quintile IV versus Quintile V Z = 0.484, *P* = 0.628. *AKI* acute kidney injury, *AUC ROC* area under the receiver operating characteristic curve, *CI* confidence interval, *Cys C* cystatin C, *FT3* free triiodothyronine

**Table 5:** Detection of AKI using Cys C by quintiles of TT3

	Total AKI^a^				Established AKI^b^				Later-onset AKI^c^			
	AUC ROC	95%CI	Cut-off	*P*	AUC ROC	95%CI	Cut-off	*P*	AUC ROC	95%CI	Cut-off	*P*
Total	0.753 ± 0.015	0.724–0.782	1.03	< 0.001	0.797 ± 0.017	0.763–0.831	0.98	< 0.001	0.669 ± 0.022	0.626–0.712	1.03	< 0.001
I (n = 268)	0.784 ± 0.028	0.729–0.838	1.22	< 0.001	0.804 ± 0.029	0.747–0.861	1.09	< 0.001	0.651 ± 0.051	0.551–0.751	1.26	0.003
II (*n* = 268)	0.738 ± 0.033	0.672–0.803	1.03	< 0.001	0.781 ± 0.039	0.705–0.856	0.98	< 0.001	0.652 ± 0.049	0.555–0.748	1.03	0.004
III (n = 268)	0.730 ± 0.035	0.661–0.799	1.07	< 0.001	0.786 ± 0.044	0.699–0.873	0.89	< 0.001	0.657 ± 0.048	0.562–0.752	1.03	0.003
IV (n = 268)	0.708 ± 0.040	0.629–0.786	0.99	< 0.001	0.706 ± 0.055	0.597–0.814	0.93	< 0.001	0.690 ± 0.054	0.585–0.795	0.68	0.002
V (n = 267)	0.707 ± 0.038	0.633–0.781	0.79	< 0.001	0.757 ± 0.050	0.660–0.855	0.81	< 0.001	0.670 ± 0.046	0.579–0.761	0.79	0.001

^a^For Total AKI, Quintile I versus Quintile II Z = 1.063, *P* = 0.288; Quintile I versus Quintile III Z = 1.205, *P* = 0.228; Quintile I versus Quintile IV Z = 1.557, *P* = 0.120; Quintile I versus Quintile V Z = 1.631, *P* = 0.103; Quintile II versus Quintile III Z = 0.166 *P* = 0.868; Quintile II versus Quintile IV Z = 0.579,*P* = 0.563; Quintile II versus Quintile V Z = 0.616, *P* = 0.538; Quintile III versus Quintile IV Z = 0.414, *P* = 0.679; Quintile III versus Quintile V Z = 0.445, *P* = 0.656; Quintile IV versus Quintile V Z = 0.018, *P* = 0.986. ^b^For Established AKI, Quintile I versus Quintile II Z = 0.473, *P* = 0.636; Quintile I versus Quintile III Z = 0.342, *P* = 0.733; Quintile I versus Quintile IV Z = 1.576, *P* = 0.115; Quintile I versus Quintile V Z = 0.813, *P* = 0.416; Quintile II versus Quintile III Z = 0.085, *P* = 0.932; Quintile II versus Quintile IV Z = 1.112, *P* = 0.266; Quintile II versus Quintile V Z = 0.378, *P* = 0.705; Quintile III versus Quintile IV Z = 1.136, *P* = 0.256; Quintile III versus Quintile V Z = 0.435, *P* = 0.663; Quintile IV versus Quintile V Z = 0.686, *P* = 0.493. ^c^For Later-onset AKI, Quintile I versus Quintile II Z = 0.014, *P* = 0.989; Quintile I versus Quintile III Z = 0.086, *P* = 0.931; Quintile I versus Quintile IV Z = 0.525, *P* = 0.600; Quintile I versus Quintile V Z = 0.277, *P* = 0.782; Quintile II versus Quintile III Z = 0.073, *P* = 0.942; Quintile II versus Quintile IV Z = 0.521, *P* = 0.602; Quintile II versus Quintile V Z = 0.268, *P* = 0.789; Quintile III versus Quintile IV Z = 0.457, *P* = 0.648; Quintile III versus Quintile V Z = 0.196, *P* = 0.845; Quintile V versus Quintile IV Z = 0.282, *P* = 0.778. *AKI* acute kidney injury, *AUC ROC* area under the receiver operating characteristic curve, *CI* confidence interval, *Cys C* cystatin C, *TT3* total triiodothyronine

### Impact of low T3 syndrome on detecting AKI using Cys C

Table 6 shows the subgroup analysis for the impact of low T3 syndrome on detecting AKI using Cys C. The patients were further separated into two categories: 650 with low T3 syndrome (FT3 < 3.8 pmol/L, and FT4 and TSH within or lower than the normal range), and 689 without low T3 syndrome. Compared with non-low T3 syndrome patients, the AUCs of Cys C in patients with low T3 syndrome were higher (0.762 vs 0.725, 0.789 vs 0.784, and 0.669 vs 0.663, respectively) in detecting total AKI, established AKI, and later-onset AKI. Furthermore, the optimal cut-off values of Cys C for AKI detection were markedly different between low T3 syndrome and non-low T3 syndrome patients. However, similar result was observed that AUCs for discrimination ability of AKI detection by Cys C did not differ significantly between groups.

**Table 6:** Detection of AKI using Cys C in patients with and without low T3 syndrome

	Total AKI^a^				Established AKI^b^			Later-onset AKI^c^				
	AUC ROC	95%CI	Cut-off	*P*	AUC ROC	95%CI	Cut-off	P	AUC ROC	95%CI	Cut-off	*P*
Total	0.753 ± 0.015	0.724–0.782	1.03	< 0.001	0.797 ± 0.017	0.763–0.831	0.98	< 0.001	0.669 ± 0.022	0.626–0.712	1.03	< 0.001
Low T3 syndrome (*n* = 650)	0.762 ± 0.020	0.723–0.801	1.03	< 0.001	0.789 ± 0.023	0.744–0.833	1.04	< 0.001	0.669 ± 0.032	0.606–0.732	1.00	< 0.001
Without lowT3 syndrome (*n* = 689)	0.725 ± 0.023	0.681–0.769	0.78	< 0.001	0.784 ± 0.028	0.730–0.838	0.77	< 0.001	0.663 ± 0.030	0.604–0.722	0.59	< 0.001

The cohort was stratified into two groups: 650 patients with low T3 syndrome, with FT3 < 3.80 pmol/L, FT4 and TSH within or lower than the normal range, and 689 patients without low T3 syndrome. ^a^For Total AKI, Z = 1.214, *P* = 0.225. ^b^For Established AKI, Z = 0.110, *P* = 0.912. ^c^For Later-onset AKI, Z = 0.138, *P* = 0.890. *AKI* acute kidney injury, *AUC ROC* area under the receiver operating characteristic curve, *CI* confidence interval, *Cys C* cystatin C, *FT3* free triiodothyronine, *FT4* free thyroxine, *TSH* thyroid-stimulating-hormone

## Discussion

This prospective, observational study assessed the influence of thyroid function on AKI detection by serum Cys C in heterogenous ICU cohort. The major finding was that there was no significant difference in the diagnostic and predictive performance of Cys C among patients with different thyroid hormone levels. However, the optimal cut-off value of Cys C to detect AKI could be affected by thyroid function alteration in ICU patients.

Serving as a clinical routine biomarker, Cys C has been postulated to be superior to SCr for early AKI identification and outcome prediction [7, 12–14]. In the diagnostic performance testing in this study, Cys C demonstrated a good diagnostic value to detect AKI, as indicated by the AUC-ROC analysis of 0.797 for established AKI. However, the predictive ability of Cys C for detecting AKI was poor with the AUC of 0.669. Furthermore, this study indicated that several factors, including age, sex, baseline SCr levels, BUN, albumin level, and thyroid hormones were related to the Cys C concentration. Therefore, whether the performance of Cys C in detecting AKI is affected by the abovementioned variables should be verified.

Increasing evidence has demonstrated that Cys C concentration could be influenced by the thyroid hormone level and was sensitive to small alterations of thyroid function [16–18]. Fricker et al. found that patients had higher concentrations of Cys C in the hyperthyroid state and lower Cys C concentrations in the hypothyroid state, thereby confirming that the thyroid function had a major impact on Cys C level [16]. Similarly, another study involved 113 patients with Graves’ disease further proved that the eGFR by CysC levels significantly decreased with elevated FT3 and FT4 levels in patients with hyperthyroidism [28]. Furthermore, Schmid et al. illustrated the mechanism by which T3 directly stimulated the production of Cys C in vitro [17]. However, a recent prospective, observational study by Wang et al., including 446 critically ill patients hospitalized in the ICU, showed a negative relationship between Cys C and thyroid function [20]. Likewise, in the present study, Cys C concentration was negatively associated with FT3, TT3, and TT4 levels. This might be explained by the significant association between low T3 syndrome and adverse outcomes in critical illness [25, 29–31], and the positive correlation between APACHE II score and Cys C. The multiple linear regression analysis showed that both TT3 and FT3 were independently correlated with Cys C. Moreover, it is acknowledged that T3 is the biologically active thyroid hormone [32, 33]. Consequently, TT3 and FT3 were selected as the representative variables to analyze the impact of thyroid function on the diagnostic and predictive value of Cys C in detecting AKI.

It was previously reported that patients with critical illness were predisposed to thyroid function derangement, of which low T3 syndrome was the most common [30, 31]. Although the presence of thyroid dysfunction seemed not to be associated with the clinical and prognostic implication of AKI in previous report [34], some other studies documented that Cys C concentration was sensitive to small change of thyroid hormones. Therefore, alterations of thyroid function should be considered when Cys C is used for AKI detection in critically ill patients [16–18]. In this regard, Schanz et al. confirmed that thyroid function had no relevant influence on clinical practice of Cys C in risk prediction of AKI in emergency department [19]. Wang et al. also reported that thyroid function, as indicated by FT4, had no impact on the performance of Cys C in diagnosing AKI in ICU settings [20]. However, one focused only on the newly AKI, and the later centered on the established AKI, using AKIN (Acute Kidney Injury Network) criteria. Based on the KDIGO criteria, the present study not only analyzed the impact of thyroid hormones, TT3 and FT3, on the diagnostic and predictive ability of Cys C in detecting AKI, but also tested the influence produced by FT4 on AKI detection by Cys C in a larger critically ill cohort. The thyroid hormones had no statistically significant effect on Cys C in diagnosing AKI on admission to ICU, in accordance with previous findings. Meanwhile, this study further confirmed that the predictive ability of Cys C in detecting AKI was also not interfered by thyroid function. In addition, the present study showed that Cys C did not yield any significant difference between patients with and without low T3 syndrome, either for established AKI, or later-onset AKI.

It was previously observed that the optimal cut-off value of Cys C in detecting AKI increased across thyroid hormone stratification [20]. However, the similar result was not displayed in the present study. The optimal cut-off value was markedly higher in patients with low T3 syndrome, in this study, the possible reason for which might be the negative correlation between Cys C and thyroid hormone concentration. This result strongly suggested that the optimal cut-off value of Cys C to detect AKI was affected by low T3 syndrome. Therefore, the reasonable cut-off point should be established when Cys C is used to detect AKI in patients with thyroid function alteration.

The present study had some limitations. First, it assessed the impact of thyroid hormones on the ability of Cys C to detect AKI, rather than predict the stratification and outcome of AKI in the critically ill patients. However, the prognostic ability of Cys C in AKI was still uncertain. Second, it only detected the effect of the baseline thyroid function on the performance of Cys C. However, critical illness is closely associated with alteration in thyroid hormone concentration. The study could not exclude that the alteration of thyroid function after admission might have an influence on Cys C in detecting AKI.

## Conclusions

This prospective, observational study found that thyroid function alteration, including low T3 syndrome, might not exert a statistically significant impact on the performance of Cys C in diagnosing and predicting AKI in ICU patients. However, the optimal cut-off value of Cys C to detect AKI could be affected by thyroid function.

## Additional file

Additional file 1: **Table S1.** Detection of AKI using Cys C by quintiles of FT4. (DOCX 20 kb)

## Abbreviations

95% CI: 95% confidence interval
AKI: Acute kidney injury
APACHE II: Acute physiology and chronic health evaluation II score
AUC: Area under the receiver operating characteristic curve
BMI: Body mass index
BUN: Blood urea nitrogen
CAD: Coronary artery disease
CHD: Chronic heart failure
CKD: Chronic Kidney Disease
EPI: Epidemiology Collaboration
Cys C: Cystatin C
DM: Diabetes mellitus
eGFR: Estimated glomerular filtration rate
ESRD: End-stage renal disease
FT3: Free triiodothyronine
FT4: Free thyroxine
HTN: Hypertension
ICU: Intensive care unit
n: Sample size
ROC: Receiver operating characteristic curve
RRT: Renal replacement therapy
SCr: Serum creatinine
TSH: Thyroid-stimulating-hormone
TT3: Total triiodothyronine
TT4: Total thyroxine
UP: Urine production first 24 h after admission
