# Supplementary material for: Impact of thyroid function on cystatin C in detecting acute kidney injury: a prospective, observational study
Source: BMC Nephrol. 2019 Feb 6;20:41. doi: 10.1186/s12882-019-1201-9 (PMC6364411; doi:10.1186/s12882-019-1201-9)
Supplement: Supplementary file 1 — Table S1. Detection of AKI using Cys C by quintiles of FT4. (DOCX 20 kb) [file 12882_2019_1201_MOESM1_ESM.docx]

**Additional file 1**

**Table S1 Detection of AKI using Cystatin C by quintiles of FT4**

|  | **Total AKI^a^** | | | | **Established AKI^b^** | | | | **Later-onset AKI^c^** | | | |
| --- | --- | --- | --- | --- | --- | --- | --- | --- | --- | --- | --- | --- |
|  | **AUC ROC** | **95%CI** | **Cut-off** | **P** | **AUC ROC** | **95%CI** | **Cut-off** | **P** | **AUC ROC** | **95%CI** | **Cut-off** | **P** |
| **Total** | 0.753 ± 0.015 | 0.724 - 0.782 | 1.03 | <0.001 | 0.797 ± 0.017 | 0.763 - 0.831 | 0.98 | <0.001 | 0.669 ± 0.022 | 0.626 - 0.712 | 1.03 | <0.001 |
| **I (268)** | 0.741 ± 0.032 | 0.677 - 0.805 | 1.13 | <0.001 | 0.791 ± 0.039 | 0.715 - 0.868 | 1.13 | <0.001 | 0.644 ± 0.047 | 0.553 - 0.736 | 0.62 | 0.005 |
| **II (268)** | 0.761 ± 0.034 | 0.695 - 0.828 | 1.02 | <0.001 | 0.778 ± 0.039 | 0.700 - 0.855 | 0.93 | <0.001 | 0.699 ± 0.053 | 0.596 - 0.803 | 0.85 | <0.001 |
| **III (268)** | 0.743 ± 0.033 | 0.679 - 0.806 | 0.76 | <0.001 | 0.801 ± 0.038 | 0.726 - 0.876 | 0.83 | <0.001 | 0.661 ± 0.045 | 0.574 - 0.749 | 0.75 | 0.002 |
| **IV (268)** | 0.789 ± 0.033 | 0.724 - 0.854 | 0.96 | <0.001 | 0.841 ± 0.038 | 0.767 - 0.916 | 1.14 | <0.001 | 0.701 ± 0.051 | 0.602 - 0.800 | 0.96 | <0.001 |
| **V (267)** | 0.731 ± 0.035 | 0.662 - 0.799 | 1.03 | <0.001 | 0.778 ± 0.039 | 0.701 - 0.855 | 1.03 | <0.001 | 0.638 ± 0.053 | 0.534 - 0.742 | 0.97 | 0.006 |

^a^For Total AKI, Quintile I versus Quintile II Z = 0.428, P = 0.668; Quintile I versus Quintile III Z = 0.044, P = 0.965; Quintile I versus Quintile IV Z = 1.044, P = 0.296; Quintile I versus Quintile V Z = 0.211, P = 0.833; Quintile II versus Quintile III Z = 0.380, P = 0.704; Quintile II versus Quintile IV Z = 0.591,P = 0.555; Quintile II versus Quintile V Z =0.615, P = 0.539; Quintile III versus Quintile IV Z = 0.986, P = 0.324; Quintile III versus Quintile V Z = 0.249, P = 0.803; Quintile IV versus Quintile V Z = 1.206, P = 0.228. ^b^For Established AKI, Quintile I versus Quintile II Z = 0.236, P = 0.814; Quintile I versus Quintile III Z = 0.184, P = 0.854; Quintile I versus Quintile IV Z =0.918, P = 0.358; Quintile I versus Quintile V Z = 0.236, P = 0.814; Quintile II versus Quintile III Z = 0.422, P = 0.672; Quintile II versus Quintile IV Z = 1.157, P = 0.247; Quintile II versus Quintile V Z = 0.000, P = 1.000; Quintile III versus Quintile IV Z = 0.744, P = 0.457; Quintile III versus Quintile V Z = 0.422, P = 0.673; Quintile IV versus Quintile V Z = 1.157, P = 0.247. ^c^For Later-onset AKI, Quintile I versus Quintile II Z = 0.776, P = 0.438; Quintile I versus Quintile III Z = 0.261, P = 0.794; Quintile I versus Quintile IV Z = 0.822, P = 0.411; Quintile I versus Quintile V Z = 0.085, P = 0.933; Quintile II versus Quintile III Z = 0.547; P = 0.585; Quintile II versus Quintile IV Z = 0.027, P = 0.978; Quintile II versus Quintile V Z = 0.814, P = 0.416; Quintile III versus Quintile IV Z = 0.588, P = 0.556; Quintile III versus Quintile V Z = 0.331, P = 0.741; Quintile IV versus Quintile V Z = 0.857, P = 0.392. AKI acute kidney injury; AUC ROC, area under the receiver operating characteristic curve; CI, confidence interval; Cys C, cystatin C; FT4, free thyroxine;.
